# Longitudinal kidney outcomes and safety after finerenone initiation in patients with non-diabetic chronic kidney disease: a single-center retrospective cohort study

**DOI:** 10.3389/fmed.2026.1959259

**Published:** 2026-10-19

**Authors:** Xuejuan Sun, Juan Pei, Xing Chen, Zhi Wang, Lijing Yao, Peijin Bai, Zhiwen Yan, Yinan Li, Leping Shao

**Affiliations:** 1 Department of Nephrology (Fujian Provincial Clinical Research Center for Glomerular Nephritis), The First Affiliated Hospital of Xiamen University, School of Medicine, Xiamen University, Xiamen, China; 2 The School of Clinical Medicine, Fujian Medical University, Fuzhou, China; 3 Department of Pharmacy, The First Affiliated Hospital of Xiamen University, School of Medicine, Xiamen University, Xiamen, China

**Keywords:** 24 h urinary protein, eGFR slope, finerenone, non-diabetic chronic kidney disease, real-world study, urinary albumin-to-creatinine ratio

## Abstract

**Background:**

Finerenone, a non-steroidal mineralocorticoid receptor antagonist, has demonstrated renoprotective effects in randomized controlled trials. However, real-world evidence, particularly in heterogeneous and non-diabetic chronic kidney disease (CKD) populations, remains limited.

**Methods:**

This was a single-center, retrospective, real-world cohort study of adults with non-diabetic CKD who initiated finerenone. Longitudinal changes in urinary albumin-to-creatinine ratio (UACR) and 24 h urinary protein (24 hUP) were analyzed using covariate-adjusted gamma-log generalized estimating equation (GEE) models with an autoregressive correlation structure of order 1 [AR(1)] and robust standard errors, whereas eGFR trajectories were evaluated using a covariate-adjusted segmented linear mixed-effects model. The UACR and 24 hUP models were adjusted for age, sex, RASi use, SGLT−2i use, and immunosuppressant use; the eGFR model additionally included baseline UACR category. Subgroup heterogeneity was assessed using interaction tests, with Benjamini–Hochberg false discovery rate correction.

**Results:**

Among 121 patients, finerenone initiation was associated with reductions in UACR and 24 hUP. The adjusted changes in UACR were −48.6% at Month 12 (95% CI, −60.1% to −33.8%; *P* < 0.001) and −35.4% at Month 24 (95% CI, −63.4% to 14.0%; *P* = 0.132). The corresponding adjusted changes in 24 hUP were −34.5% (95% CI, −47.3% to −18.6%; *P* < 0.001) and −34.9% (95% CI, −56.6% to −2.4%; *P* = 0.038), respectively. The annualized eGFR slope attenuated from −6.51 mL/min/1.73 m^2^/year (95% CI, −9.70 to −3.31) before finerenone initiation to −1.44 mL/min/1.73 m^2^/year (95% CI, −3.52 to 0.64) after initiation, corresponding to a slope improvement of 5.07 mL/min/1.73 m^2^/year (95% CI, 1.86 to 8.27; *P* = 0.002). No serious adverse events were documented during follow-up; hyperkalemia occurred in 4.96% of patients, and neither hyperkalemia nor hypotension prompted treatment discontinuation.

**Conclusion:**

In this real-world cohort, finerenone was associated with reductions in UACR and 24 hUP and attenuation of eGFR decline, with infrequent observed hyperkalemia and hypotension during the available follow-up.

## Introduction

Chronic kidney disease (CKD) affects an estimated 850 million people worldwide, and although diabetes is a leading cause, 50%–80% of cases arise from non-diabetic etiologies (1–5). Regardless of underlying cause, disease progression is closely linked to the severity of proteinuria, with reductions in albuminuria consistently associated with a lower risk of kidney failure (6).

Renin–angiotensin system inhibitors (RASi) and sodium–glucose cotransporter-2 inhibitors (SGLT-2i) remain the cornerstone of nephroprotective therapy and have meaningfully improved renal outcomes in CKD (7, 8). In non-diabetic CKD specifically, management is further individualized according to etiology, with immunosuppressive therapy reserved for selected patients with immune-mediated kidney disease (7). Nonetheless, many patients continue to experience persistent proteinuria and progressive kidney function decline despite these measures, highlighting a continued need for additional therapeutic options that act through complementary pathways (9). Overactivation of the mineralocorticoid receptor—driving sodium/fluid retention and profibrotic, proinflammatory signaling—has emerged as one such pathway (10, 11).

Finerenone is a selective nonsteroidal mineralocorticoid receptor antagonist that has been shown to reduce albuminuria and slow kidney disease progression in patients with diabetic CKD in the FIDELIO-DKD and FIGARO-DKD trials (12–16). Growing evidence now supports its benefit beyond diabetic kidney disease: the recently completed FIND-CKD trial demonstrated that finerenone significantly slowed estimated glomerular filtration rate (eGFR) decline, the trial's primary outcome, and reduced the risk of a composite kidney-cardiovascular outcome as a secondary endpoint, in patients with non-diabetic CKD (17). Real-world experience in this population, however, remains limited to a small number of single-center studies with modest sample sizes and follow-up of 12 months or less, leaving longer-term effectiveness and safety—particularly in patients receiving concomitant immunosuppression—incompletely characterized (18, 19).

We conducted a retrospective real-world study with a longer follow-up and larger sample size than previously reported, evaluating the effectiveness and safety of finerenone in a broader population of patients with non-diabetic CKD, including those receiving concomitant immunosuppressive therapy.

## Methods

### Study design and patients

This was a single-center, real-world, retrospective cohort study. Adult patients with chronic kidney disease (CKD) who initiated finerenone treatment at the First Affiliated Hospital of Xiamen University between January 2023 and December 2025 were screened.

The use of finerenone in this study population (non-diabetic CKD) was considered off-label. Its use was reviewed and approved by the institutional Drug and Therapeutics Committee, based on clinical judgment and individual patient needs. As this was a retrospective observational study using de-identified clinical data, the requirement for individual informed consent was waived by the institutional ethics committee. The study protocol, including the off-label use of finerenone, was approved by the institutional ethics committee in accordance with the Declaration of Helsinki.

Inclusion criteria required: (1) confirmed CKD diagnosis per KDIGO 2012 guidelines (20); (2) regular finerenone use for ≥90 days; (3) age 18–80 years at finerenone initiation; (4) baseline eGFR ≥20 mL/min/1.73 m^2^ [eGFR was calculated from serum creatinine using the 2009 Chronic Kidney Disease Epidemiology Collaboration (CKD-EPI) creatinine equation] and albuminuria (UACR ≥30 mg/g); (5) stable background therapy (RASi and/or SGLT-2i, and other etiology-specific treatments such as immunosuppressive agents when applicable) for ≥90 days prior to finerenone initiation, with no significant treatment changes during this period, and finerenone added at physician discretion for persistent proteinuria despite standard-of-care therapy.

Exclusion criteria included: (1) type 1 or type 2 diabetes, or baseline HbA1c ≥6.5%; (2) baseline hyperkalemia (serum potassium ≥5.0 mmol/L); (3) substantial missing baseline or follow-up data (≥20% missing for key variables); (4) renal replacement therapy (dialysis or transplantation); (5) concurrent participation in another interventional trial affecting renal outcomes; (6) Child-Pugh class C cirrhosis, severe heart failure (NYHA class IV, or LVEF <50% with echocardiographic evidence of diastolic dysfunction), or uncontrolled hypertension (SBP ≥180 mmHg and/or DBP ≥110 mmHg despite antihypertensive therapy).

All patients who initiated finerenone during the study period were consecutively screened according to predefined eligibility criteria. Because this was a rolling-enrollment retrospective cohort, patients entered the cohort at different calendar time points and therefore had variable potential follow-up durations. The ≥90-day requirement was applied to ensure sufficient treatment exposure and longitudinal observation for assessment of changes in proteinuria and kidney function. Among patients considered for eligibility, 36 did not meet or could not be confirmed to meet this requirement: 32 had no documented follow-up at our institution within 90 days after finerenone initiation, and 4 initiated finerenone <90 days before the database cutoff. For the former group, whether finerenone was discontinued or continued at another institution could not be reliably determined from the retrospective records. After application of the eligibility criteria, 121 patients were included in the final analysis. The patient selection process is summarized in Supplementary Figure S1.

### Clinical data collection

Standardized clinical data were collected for all participants, including demographic and clinical characteristics: sex, age (years), primary disease diagnosis, comorbidities, time of finerenone initiation, and medication dosage (mg/day), concomitant use of RASi, SGLT-2i, and immunosuppressive therapies (including corticosteroids and other immunosuppressants such as cyclosporine, tacrolimus, mycophenolate mofetil, and azathioprine), or biologics (e.g., rituximab, belimumab, telitacicept, if applicable).

Longitudinal measurements were obtained at predefined follow-up time points, including renal and metabolic parameters: UACR (mg/g), 24 hUP (mg/24 h), serum creatinine (μmol/L), serum potassium (mmol/L), eGFR (mL/min/1.73 m^2^), liver function tests, and creatine kinase.

To enable estimation of the pre-treatment eGFR slope, all available serum creatinine and eGFR measurements recorded within the 12 months prior to finerenone initiation were also retrospectively collected from the electronic medical record, in addition to the baseline and post-treatment measurements described above.

Hyperkalemia severity was graded based on serum potassium levels as follows: Grade 0: <5.0 mmol/L, Grade 1: 5.0–5.4 mmol/L, Grade 2: 5.5–5.9 mmol/L, Grade 3: ≥6.0 mmol/L. Hyperkalemia was clinically defined as serum potassium ≥5.5 mmol/L.

Baseline was defined as the date of first finerenone prescription. Follow-up assessments were scheduled at Months 1, 3, 6, 9, 12, 15, 18, 21, and 24, using a prespecified time window (±15 days for Month 1; ±30 days thereafter); when multiple measurements fell within a window, the value closest to the target date was used. Only patients with available data at each time point were included in the corresponding analysis, without imputation for missing values.

### Efficacy evaluation

The proteinuria thresholds defining complete remission (CR) and partial remission (PR) were adapted from remission and response criteria widely used in guidelines and clinical studies of proteinuric glomerular diseases (7, 21, 22), and applied to the overall CKD cohort in the present study. CR was defined as 24 h urinary protein (24 hUP) < 300 mg/24 h, with no ≥30% decline in eGFR from baseline and an annualized eGFR decline of <5 mL/min/1.73 m^2^/year. PR was defined as a ≥50% reduction in 24 hUP from baseline without meeting the CR criteria. CKD progression was defined as either a sustained ≥30% decline in eGFR from baseline or an annualized eGFR decline of ≥5 mL/min/1.73 m^2^/year (23).

### Outcomes

The three principal efficacy outcomes were: (1) longitudinal percentage change in UACR from baseline; (2) longitudinal percentage change in 24 hUP from baseline; and (3) change in eGFR trajectory after finerenone initiation, quantified by the pre-treatment eGFR slope, post-treatment eGFR slope, and the difference between the two slopes. Complete remission, partial remission, CKD progression, blood pressure, serum potassium, and adverse events were evaluated as additional clinical and safety outcomes.

Exploratory subgroup analyses of UACR and 24 hUP were performed according to baseline eGFR (<60 vs. ≥60 mL/min/1.73 m^2^), immunosuppressant use (no vs. yes), RASi use (no vs. yes), and SGLT-2i use (no vs. yes). Exploratory subgroup analyses of eGFR slopes additionally included baseline UACR (<300 vs. ≥300 mg/g).

### Safety assessment

Adverse events (AEs) and serious adverse events (SAEs) were systematically assessed. SAEs were defined as events that were life-threatening, resulted in death, required unplanned hospitalization or prolongation of an existing hospitalization, or caused persistent or permanent organ dysfunction. Safety assessments included hyperkalemia (serum potassium ≥5.5 mmol/L), hypotension (systolic blood pressure <90 mmHg or diastolic blood pressure <60 mmHg), cardiovascular and cerebrovascular events, hepatotoxicity, and other clinically significant AEs, including breast hyperplasia and gouty arthritis.

### Statistical analysis

Continuous variables were assessed for distributional shape and are presented as mean ± standard deviation or median (interquartile range), as appropriate. Categorical variables are presented as numbers and percentages. All longitudinal analyses used available observations within the prespecified visit windows; missing follow-up values were not imputed.

Covariates were selected based on clinical relevance and model parsimony rather than data-driven variable selection. Age and sex were included as fundamental demographic prognostic factors, whereas baseline RASi use, SGLT-2i use, and immunosuppressant use were included because these treatments could influence proteinuria and kidney-function trajectories. Baseline UACR category was additionally included in the eGFR model as an indicator of baseline kidney disease severity.

Longitudinal changes in UACR and 24 hUP were analyzed using multivariable GEE models with a gamma distribution and log link. Time was modeled as a categorical variable, with baseline as the reference. An AR(1) working correlation structure was specified, and robust sandwich standard errors were used for inference. Overall-cohort models were adjusted for age, sex, baseline RASi use, baseline SGLT-2i use, and baseline immunosuppressant use. Model coefficients were exponentiated and expressed as adjusted percentage changes from baseline with 95% confidence intervals. A global Wald test assessed the overall effect of time.

eGFR trajectories were analyzed using a multivariable segmented linear mixed-effects model with a knot at finerenone initiation. Time was expressed in years, allowing direct interpretation of model coefficients as annualized eGFR slopes. The model included patient-specific random intercepts and random slopes for time and was fitted using restricted maximum likelihood. Fixed-effect inference and 95% confidence intervals were based on the Satterthwaite approximation. The model estimated the annualized pre-treatment slope, annualized post-treatment slope, and slope improvement, defined as the post-treatment slope minus the pre-treatment slope. The eGFR model was adjusted for age, sex, baseline UACR category (<300 vs. ≥300 mg/g), baseline RASi use, baseline SGLT-2i use, and baseline immunosuppressant use.

Sensitivity analyses were performed to assess the potential influence of kidney disease etiology and baseline disease severity. Kidney disease etiology was categorized as IgA nephropathy, membranous nephropathy, or other etiologies to avoid unstable estimates from sparsely represented diagnostic categories. For UACR and 24 hUP, sensitivity models additionally adjusted for baseline eGFR and kidney disease etiology. For eGFR, the mixed-effects model was additionally adjusted for kidney disease etiology; in a separate sensitivity model, categorical baseline UACR was replaced by continuous log-transformed baseline UACR. Because baseline proteinuria differed substantially according to immunosuppressant use, complementary post-baseline gamma-log GEE models were also fitted. These models excluded the baseline outcome observation and explicitly adjusted for the corresponding log-transformed baseline proteinuria measurement, baseline eGFR, kidney disease etiology, age, sex, RASi use, and SGLT-2i use. Formal time-by-immunosuppression interaction tests assessed whether post-baseline trajectories differed between immunosuppression groups.

To assess potential differential follow-up at Month 24, the baseline comparison was restricted to patients who were calendar-eligible for the Month-24 visit. Calendar eligibility was defined as having sufficient potential follow-up to enter the prespecified Month-24 visit window (730 ± 30 days) before the database cutoff of December 31, 2025; accordingly, patients with a baseline date on or before January 31, 2024 were considered calendar-eligible. Among these patients, the Month-24 key outcome available group comprised those with at least one available UACR, 24 h urinary protein, or scheduled eGFR measurement within the Month-24 visit window, whereas the missing despite Month-24 eligibility group comprised those with none of these key outcomes available within that window. Baseline characteristics were compared between these two groups to evaluate potential differential follow-up and attrition bias.

For each subgroup analysis, the corresponding stratifying variable was omitted from the covariate set and an interaction term was added. For UACR and 24 hUP, the global time-by-subgroup interaction tested whether the longitudinal trajectory differed between subgroup levels across the nine post-baseline visits. For eGFR, the interaction test assessed heterogeneity in slope improvement between subgroup levels. Benjamini-Hochberg FDR correction was applied separately to the interaction-test families for UACR (four subgroup factors), 24 hUP (four subgroup factors), and eGFR slope (five subgroup factors). Pointwise within-subgroup *P* values and the three principal efficacy-outcome analyses were not included in these FDR families. Serum potassium and blood pressure were analyzed using Gaussian-identity GEE models with time as a categorical variable and baseline as the reference, yielding estimated marginal means at each visit. All tests were two-sided, with *P* < 0.05 considered statistically significant. Analyses were performed using R version 4.3.

## Results

### Study population and baseline characteristics

A total of 121 eligible patients with non-diabetic CKD who initiated finerenone were included in the analysis (total cohort); 91 patients who did not receive concomitant immunosuppressive therapy constituted the non-immunosuppressive subgroup, and 30 patients receiving immunosuppressive therapy constituted the immunosuppressive subgroup. Baseline characteristics are summarized in Table 1. The total cohort had a mean age of 45.2 ± 12.7 years, with 51.2% female. Mean eGFR was 80.1 ± 28.5 mL/min/1.73 m^2^, and median 24 hUP and UACR were 1,021.0 mg/24 h (IQR 453.2–1,770.0) and 586.0 mg/g (IQR 259.3–1,329.3), respectively. Compared with the non-immunosuppressive subgroup, patients in the immunosuppressive subgroup had higher baseline proteinuria and albuminuria (24 hUP 2,852.2 vs. 751.5 mg/24 h; UACR 1,332.5 vs. 431.6 mg/g), consistent with the underlying disease profile of patients selected for immunosuppressive treatment. IgA nephropathy (42.1%) and membranous nephropathy (28.1%) were the most common etiologies. Most patients received RASi (82.6%) and/or SGLT-2i (45.5%), with 90.1% receiving at least one guideline-recommended background therapy; immunosuppressive therapy was used in 24.8% of patients, and no patients received biologic agents.

**Table 1:** Demographic and clinical characteristics of patients at baseline.

Characteristics	Total cohort (*N* = 121)	Non-immunosuppressive therapy subgroup (*N* = 91)	Immunosuppressive therapy subgroup (*N* = 30)	P
Demographics				
Age (years)	45.21 ± 12.67	45.18 ± 12.06	45.30 ± 14.59	0.967
Female	62 (51.2%)	49 (53.8%)	13 (43.3%)	0.318
Laboratory tests or examinations				
eGFR, mL/min/1.73 m^2^	80.09 ± 28.47	79.29 ± 29.30	82.52 ± 29.10	0.571
*eGFR, mL/min/1.73 m^2^, N%*				0.337
<30	4 (3.3%)	3 (3.3%)	1 (3.3%)	
30–<60	28 (23.1%)	24 (26.4%)	4 (13.3%)	
≥60	89 (73.6%)	64 (70.3%)	25 (83.3%)	
24 hUP, mg/24 h	1,021.00 (453.24–1,770.00)	751.50 (357.76–1,347.25)	2,852.24 (1,327.75–4,054.93)	0.002
UACR, mg/g	586.00 (259.32–1,329.30)	431.57 (206.10–981.21)	1,332.52 (496.32–2,017.18)	0.028
*UACR, mg/g, N%*				0.028
UACR <300 mg/g, N%	40 (33.1%)	38 (41.76%)	2 (6.7%)	
UACR ≥300 mg/g, N%	81 (66.9%)	53 (58.24%)	28 (93.3%)	
sK + , mmol/L	4.14 (3.96–4.37)	4.14 (3.95–4.41)	4.17 (3.98–4.35)	0.949
ALB, g/L	39.58 ± 6.20	40.80 ± 5.05	35.95 ± 7.79	0.003
ALT, U/L	18.3 (14.0–25.7)	18.40 (14.20, 25.80)	18.10 (14.55, 24.20)	0.432
AST, U/L	22.0 (17.4–26.6)	22.2 (18.45–26.9)	20.15 (14.13–24.30)	0.689
CK, U/L	81.5 (60.0–123.5)	85 (63–129)	72 (51–101)	0.079
systolic blood pressure (mmHg)	128.00 (120.00–142.00)	131.00 (121.00–148.00)	124.00 (120.00–129.00)	0.095
diastolic blood pressure (mmHg)	78.00 (72.00–86.00)	84.00 (76.50–87.50)	79.50 (71.75–86.00)	0.613
Dosage Regimen (mg/d)				
Initial Dose				1.000
10 mg	115 (95.0%)	86 (94.5%)	29 (96.7%)	
20 mg	6 (5.0%)	5 (5.5%)	1 (3.3%)	
Dose Adjustment				1.000
Uptitration (10 mg/day to 20 mg/day)	8 (6.6%)	6 (6.6%)	2 (6.7%)	
Downtitration (20 mg/day to 10 mg/day)	1 (0.8%)	1 (1.1%)	0 (0.0%)	
Kidney diseases (etiological diagnosis)				0.014
IgA nephropathy	51 (42.1%)	43 (47.3%)	8 (26.67%)	
Membranous nephropathy	34 (28.1%)	20 (22.0%)	14 (46.7%)	
FSGS	2 (1.7%)	1 (1.1%)	1 (3.3%)	
Mesangial proliferative glomerulonephritis	3 (2.5%)	3 (3.3%)	0 (0.0%)	
Focal proliferative glomerulonephritis	1 (0.8%)	1 (1.1%)	0 (0.0%)	
Hypertensive renal damage	1 (0.8%)	1 (1.1%)	0 (0.0%)	
Lupus nephritis	1 (0.8%)	0 (0.0%)	1 (3.3%)	
Hepatitis B virus-associated glomerulonephritis	1 (0.8%)	0 (0.0%)	1 (3.3%)	
Henoch-Schönlein purpura nephritis	1 (0.8%)	0 (0.0%)	1 (3.3%)	
Polycystic kidney disease	2 (1.7%)	2 (2.2%)	0 (0.0%)	
Chronic nephritis	15 (12.4%)	13 (14.3%)	2 (6.7%)	
Primary nephrotic syndrome*	4 (3.3%)	2 (2.2%)	2 (6.7%)	
Etiology unknown	5 (4.1%)	5 (5.5%)	0 (0.0%)	
Comorbidities				
Hypertension	50 (41.3%)	38 (41.8%)	12 (40.0%)	0.865
Sjögren's syndrome	1 (0.8%)	1 (1.1%)	0 (0.0%)	1.000
Hashimoto's thyroiditis	1 (0.8%)	0 (0.0%)	1 (3.3%)	0.248
Ureteral calculi	1 (0.8%)	1 (1.1%)	0 (0.0%)	1.000
Heart valve disease	1 (0.8%)	1 (1.1%)	0 (0.0%)	1.000
Isolated kidney (right side)	1 (0.8%)	1 (1.1%)	0 (0.0%)	1.000
Coronary heart disease	1 (0.8%)	1 (1.1%)	0 (0.0%)	1.000
Nutcracker syndrome	1 (0.8%)	0 (0.0%)	1 (3.3%)	0.248
Hypothyroidism	1 (0.8%)	1 (1.1%)	0 (0.0%)	1.000
Kidney tumor	1 (0.8%)	1 (1.1%)	0 (0.0%)	1.000
Atrophy of the right kidney	1 (0.8%)	1 (1.1%)	0 (0.0%)	1.000
Concomitant medications				
RASi	100 (82.6%)	73 (80.2%)	27 (90.0%)	0.220
SGLT-2i	55 (45.5%)	39 (42.9%)	16 (53.3%)	0.318
Immunosuppressants	30 (24.8%)	0 (0.0%)	30 (100%)	
Glucocorticoids, Cyclophosphamide	1 (0.8%)	0 (0.0%)	1 (3.3%)	
Glucocorticoids,Tacrolimus	1 (0.8%)	0 (0.0%)	1 (3.3%)	
Cyclosporine	1 (0.8%)	0 (0.0%)	1 (3.3%)	
Cyclophosphamide	3 (2.5%)	0 (0.0%)	3 (10.0%)	
Mycophenolate mofetil	7 (5.8%)	0 (0.0%)	7 (23.3%)	
Tacrolimus	11 (9.1%)	0 (0.0%)	11 (36.7%)	
Tacrolimus, Cyclophosphamide	6 (5.0%)	0 (0.0%)	6 (20.0%)	

No patients in this cohort received biologic therapy throughout the study.

* Primary nephrotic syndrome was defined based on clinical presentation; kidney biopsy was not performed in these patients.

eGFR, estimated glomerular filtration rate; 24 hUP, 24 h urinary protein; UACR, urinary albumin-to-creatinine ratio; sK+, serum potassium; ALB, albumin; ALT, alanine aminotransferase; AST, aspartate aminotransferase; CK, creatine kinase; FSGS, focal segmental glomerulosclerosis; RASi, renin-angiotensin system inhibitor; SGLT-2i, sodium-glucose cotransporter-2 inhibitor.

The median observed follow-up duration was 434 days (IQR, 278–604 days), defined from finerenone initiation to the last documented clinical encounter. Given staggered enrollment, fewer patients had sufficient calendar time to complete later prespecified visits. We therefore differentiated two groups: patients with missing outcome data despite reaching the corresponding visit window, and those who had not yet reached the window at database cutoff (Supplementary Table S7). At Month 24, 46 of 121 patients were calendar-eligible for the visit window; of these, 20 had available key outcome data and 26 had missing key outcomes. The remaining 75 patients had not reached the Month-24 window by database closure. A baseline comparison between patients with and without available Month-24 key outcome data among the 46 calendar-eligible patients is presented in Supplementary Table S8.

### Finerenone dosage

Most patients (95.0%) initiated finerenone at 10 mg/day, reflecting cautious dose selection given the elevated risk of hyperkalemia in CKD (Table 1). Dose uptitration to 20 mg/day occurred in 6.6% of patients during follow-up, typically within the first 1–3 months after confirming stable potassium and renal function; downtitration occurred in one patient. A similar pattern was observed across subgroups.

### Primary efficacy outcomes

#### Urinary albumin-to-creatinine ratio

UACR decreased early and substantially after treatment initiation (Figure 1A). Adjusted changes were −22.1% at Month 1 (95% CI, −31.2% to −11.9%; *P* < 0.001), −41.5% at Month 3 (95% CI, −50.9% to −30.2%; *P* < 0.001), and −48.6% at Month 12 (95% CI, −60.1% to −33.8%; *P* < 0.001). Reductions remained statistically significant through Month 21. At Month 24, UACR remained numerically lower than baseline (−35.4%; 95% CI, −63.4% to 14.0%), but the estimate was imprecise and no longer statistically significant (*P* = 0.132).

**Figure 1:**
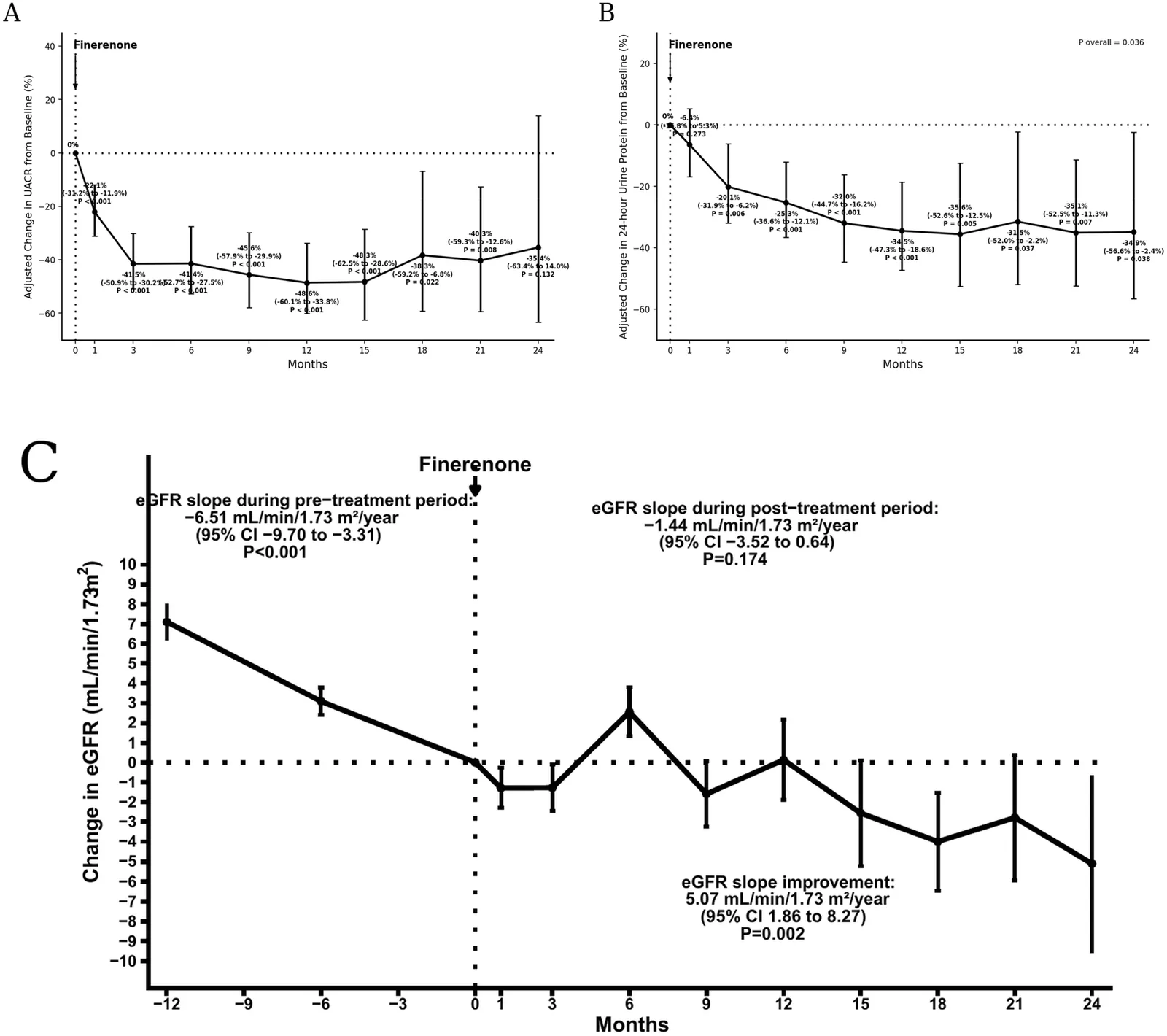
Longitudinal changes in urinary albumin-to-creatinine ratio, 24 h urinary protein, and eGFR after finerenone initiation. **(A)** Adjusted percentage change in urinary albumin-to-creatinine ratio (UACR) from baseline. **(B)** Adjusted percentage change in 24 h urinary protein (24 hUP) from baseline. **(C)** Mean change in estimated glomerular filtration rate (eGFR) from baseline and adjusted annualized eGFR slopes before and after finerenone initiation. Error bars represent 95% confidence intervals in panels **A** and **B** and standard errors in panel **C**. The vertical dotted line indicates finerenone initiation, and the horizontal dotted line indicates no change from baseline.

#### 24 h urinary protein

In the adjusted gamma-log GEE model, 24 hUP decreased progressively after finerenone initiation (Figure 1B). The reduction was not statistically significant at Month 1 (−6.4%; 95% CI, −16.8% to 5.3%; *P* = 0.273), but became significant at Month 3 (−20.1%; 95% CI, −31.9% to −6.2%; *P* = 0.006) and remained significant through Month 24. Adjusted changes were −34.5% at Month 12 (95% CI, −47.3% to −18.6%; *P* < 0.001) and −34.9% at Month 24 (95% CI, −56.6% to −2.4%; *P* = 0.038). The global time effect was significant (*P* overall = 0.036).

### eGFR trajectory

The adjusted annualized eGFR slope was −6.51 mL/min/1.73 m^2^/year during the 12-month pre-treatment period (95% CI, −9.70 to −3.31; *P* < 0.001) and −1.44 mL/min/1.73 m^2^/year after finerenone initiation (95% CI, −3.52 to 0.64; *P* = 0.174) (Figure 1C). The estimated slope improvement was 5.07 mL/min/1.73 m^2^/year (95% CI, 1.86 to 8.27; *P* = 0.002), indicating a statistically significant attenuation in the rate of eGFR decline after treatment initiation.

### Sensitivity analyses

After additional adjustment for baseline eGFR and kidney disease etiology, the adjusted UACR changes were −47.2% at Month 12 (95% CI, −58.2% to −33.3%) and −36.8% at Month 24 (95% CI, −63.9% to 10.8%; *P* overall<0.001). The corresponding changes in 24 h urinary protein were −35.1% (95% CI, −46.6% to −21.1%) and −35.6% (95% CI, −56.7% to −4.1%; *P* overall = 0.005), respectively. Additional adjustment for kidney disease etiology had little effect on the eGFR slope improvement (5.04 mL/min/1.73 m^2^/year; 95% CI, 1.84 to 8.25; *P* = 0.002), and results were similar when categorical baseline UACR was replaced with continuous log-transformed UACR (5.30 mL/min/1.73 m^2^/year; 95% CI, 2.09 to 8.52; *P* = 0.001).

In complementary post-baseline analyses explicitly adjusting for the corresponding baseline proteinuria measurement, effect estimates were attenuated. The standardized changes at Month 12 were −23.6% for UACR (95% CI, −35.5% to −9.6%) and −11.9% for 24 h urinary protein (95% CI, −24.5% to 2.9%); the corresponding Month 24 estimates were −18.9% (95% CI, −44.9% to 19.4%) and −15.2% (95% CI, −38.1% to 16.2%), respectively. The time-by-immunosuppression interaction was not statistically significant for either UACR (*P* = 0.354) or 24 h urinary protein (*P* = 0.063). Full sensitivity-analysis results are provided in Supplementary Tables S3–S6.

### Exploratory subgroup analyses

UACR subgroup results at Months 12 and 24 are summarized in Table 2; complete estimates for all nine post-baseline visits are provided in Supplementary Table S2. Significant within-subgroup reductions were observed across several levels of baseline eGFR, immunosuppressant use, RASi use, and SGLT-2i use. The global time-by-RASi interaction remained significant after FDR correction (*P* for interaction <0.001; FDR-adjusted *P* < 0.001), indicating that the shape of the UACR trajectory differed according to baseline RASi use. Interactions for baseline eGFR, immunosuppressant use, and SGLT-2i use did not remain significant after FDR correction. Because the RASi-negative subgroup was relatively small and the pattern was not uniformly greater in one group at every visit, this interaction should be considered exploratory rather than evidence of a definitively superior response in either subgroup.

**Table 2:** Subgroup analyses of urinary albumin-to-creatinine ratio at months 12 and 24 after finerenone initiation.

Subgroup factor	Category	Month 12 adjusted change, % (95% CI)	*P* value	Month 24 adjusted change, % (95% CI)	*P* value	*P* for interaction	FDR-adjusted *P*
Baseline eGFR	<60	−42.9 (−58.8 to −20.9)	**<0** **.** **001**	−15.6 (−45.9 to 31.8)	0.456	0.092	0.184
Baseline eGFR	≥60	−51.4 (−64.7 to −33.1)	**<0** **.** **001**	−47.6 (−80.6 to 41.5)	0.202	0.092	0.184
Immunosuppressant	No	−47.4 (−60.8 to −29.3)	**<0** **.** **001**	−34.0 (−64.9 to 24.1)	0.197	0.963	0.963
Immunosuppressant	Yes	−47.2 (−62.7 to −25.3)	**<0** **.** **001**	−20.9 (−54.3 to 37.0)	0.403	0.963	0.963
RASi	No	−51.5 (−79.2 to 13.1)	0.094	−33.8 (−62.1 to 15.4)	0.146	**<0** **.** **001**	**<0** **.** **001**
RASi	Yes	−47.0 (−57.8 to −33.4)	**<0** **.** **001**	−33.4 (−63.4 to 21.0)	0.182	**<0** **.** **001**	**<0** **.** **001**
SGLT-2i	No	−46.5 (−62.1 to −24.5)	**<0** **.** **001**	−21.2 (−63.4 to 69.5)	0.541	0.208	0.278
SGLT-2i	Yes	−50.6 (−65.6 to −29.0)	**<0** **.** **001**	−63.8 (−78.1 to −40.1)	**<0** **.** **001**	0.208	0.278

Values are adjusted percentage changes from baseline with 95% CIs from gamma-log GEE models with an AR(1) working correlation structure and robust sandwich standard errors. Models were adjusted for age, sex, RASi use, SGLT-2i use, and immunosuppressant use; the stratifying variable was omitted from the corresponding subgroup model. *P* for interaction is the global time-by-subgroup interaction test using all nine post-baseline visits and therefore is not calculated from Months 12 and 24 alone. FDR adjustment used the Benjamini-Hochberg method across the four UACR interaction tests. Complete results for all visits are provided in Supplementary Table S2.

Bold values indicate statistically significant *P* values (*P* < 0.05).

eGFR slope subgroup analyses are shown in Table 3. Nominally significant within-stratum slope improvements were observed in participants with baseline eGFR <60 mL/min/1.73 m^2^, baseline eGFR ≥60 mL/min/1.73 m^2^, baseline UACR ≥300 mg/g, no immunosuppressant use, RASi use, and no SGLT-2i use. However, none of the interaction tests remained significant after FDR correction, providing no robust evidence that slope improvement differed by baseline eGFR, baseline UACR, immunosuppressant use, RASi use, or SGLT-2i use.

**Table 3:** Subgroup analyses of eGFR slopes after finerenone initiation.

Subgroup factor	Category	Pre-treatment slope, estimate (95% CI)	Post-treatment slope, estimate (95% CI)	Slope improvement, estimate (95% CI)	*P* value	*P* for interaction	FDR-adjusted *P*
Baseline eGFR	<60	−9.01 (−16.36 to −1.66)	0.38 (−4.43 to 5.18)	9.39 (2.11 to 16.67)	**0** **.** **012**	0.155	0.656
Baseline eGFR	≥60	−5.65 (−8.98 to −2.32)	−2.13 (−4.28 to 0.03)	3.52 (0.11 to 6.93)	**0** **.** **043**	0.155	0.656
Baseline UACR	<300 mg/g	−5.61 (−11.16 to −0.06)	−3.14 (−6.72 to 0.44)	2.47 (−3.07 to 8.02)	0.381	0.262	0.656
Baseline UACR	≥300 mg/g	−6.95 (−10.86 to −3.04)	−0.55 (−3.11 to 2.02)	6.40 (2.46 to 10.34)	**0** **.** **001**	0.262	0.656
Immunosuppressant	No	−5.95 (−9.58 to −2.32)	−1.16 (−3.37 to 1.05)	4.79 (1.07 to 8.51)	**0** **.** **012**	0.982	0.982
Immunosuppressant	Yes	−7.45 (−14.11 to −0.79)	−3.44 (−8.75 to 1.86)	4.00 (−2.06 to 10.06)	0.194	0.982	0.982
RASi	No	−6.93 (−14.87 to 1.01)	−3.22 (−7.92 to 1.48)	3.72 (−4.85 to 12.29)	0.392	0.877	0.982
RASi	Yes	−6.24 (−9.72 to −2.77)	−1.02 (−3.32 to 1.29)	5.23 (1.77 to 8.69)	**0** **.** **003**	0.877	0.982
SGLT-2i	No	−7.18 (−11.81 to −2.56)	−1.55 (−4.62 to 1.52)	5.63 (1.06 to 10.20)	**0** **.** **016**	0.612	0.982
SGLT-2i	Yes	−5.42 (−9.77 to −1.07)	−1.42 (−4.22 to 1.38)	4.00 (−0.41 to 8.40)	0.075	0.612	0.982

Values are annualized eGFR slopes in mL/min/1.73 m^2^/year and are presented as estimates (95% CIs). Slope improvement was defined as the post-treatment slope minus the pre-treatment slope. *P* for interaction evaluates differences in slope improvement between subgroup categories.

CI, confidence interval; FDR, false discovery rate.

Bold values indicate statistically significant *P* values (*P* < 0.05).

Subgroup analyses of 24 hUP are presented in Supplementary Table S1. Although adjusted reductions were observed in multiple subgroup levels, no time-by-subgroup interaction remained significant after FDR correction. Thus, the data did not provide robust evidence of heterogeneity in the 24 hUP response across baseline eGFR, immunosuppressant use, RASi use, or SGLT-2i use.

### Treatment response and renal outcomes

Treatment response and CKD progression in the overall cohort are summarized in Table 4. The combined CR and PR rate was 28.4% at Month 12 and 45.0% at Month 24, while CKD progression was observed in 21.0% and 25.0% of patients, respectively. Because only 20 patients had available data at Month 24, the late follow-up estimates should be interpreted cautiously. No formal comparisons across follow-up visits were performed.

**Table 4:** Treatment response and CKD progression among patients with available data at each follow-up visit (total cohort).

Time (months)	No. of patients	CR *n* (%)	PR *n* (%)	CKD progression *n* (%)
3	115	9 (7.8%)	8 (7.0%)	13 (11.3%)
6	112	15 (13.4%)	20 (17.9%)	11 (9.8%)
9	101	15 (14.9%)	22 (21.8%)	16 (15.8%)
12	81	13 (16.0%)	10 (12.3%)	17 (21.0%)
15	55	10 (18.2%)	9 (16.4%)	15 (27.3%)
18	43	8 (18.6%)	8 (18.6%)	13 (30.2%)
21	24	7 (29.2%)	5 (20.8%)	7 (29.2%)
24	20	7 (35.0%)	2 (10.0%)	5 (25.0%)

Complete remission (CR) was defined as 24 h urinary protein (24 hUP) <300 mg/24 h, with no ≥30% decline in eGFR from baseline and an annualized eGFR decline of <5 mL/min/1.73 m^2^/year. Partial remission (PR) was defined as a ≥50% reduction in 24 hUP from baseline without meeting the CR criteria. CKD progression was defined as either a sustained ≥30% decline in eGFR from baseline or an annualized eGFR decline of ≥5 mL/min/1.73 m^2^/year. The denominator for all percentages at each visit was the number of patients shown in the “No. of patients” column. CR and PR were mutually exclusive; CKD progression was assessed independently and could overlap with PR. Analyses were based on available cases without imputation, and no formal comparisons across follow-up visits were performed. The number of available observations decreased at later visits because patients had different potential follow-up durations in this rolling-enrollment cohort.

### Safety and adverse events

During the observed follow-up, no participant experienced an SAE. Clinically defined hyperkalemia (serum potassium ≥5.5 mmol/L) occurred in 6 of 121 participants (4.96%) and was managed with short-course calcium polystyrene sulfonate and dietary counseling, without treatment discontinuation. Estimated marginal means of serum potassium and blood pressure remained within clinically acceptable ranges throughout follow-up, and no hypotension-related treatment discontinuations occurred (Figure 2).

**Figure 2:**
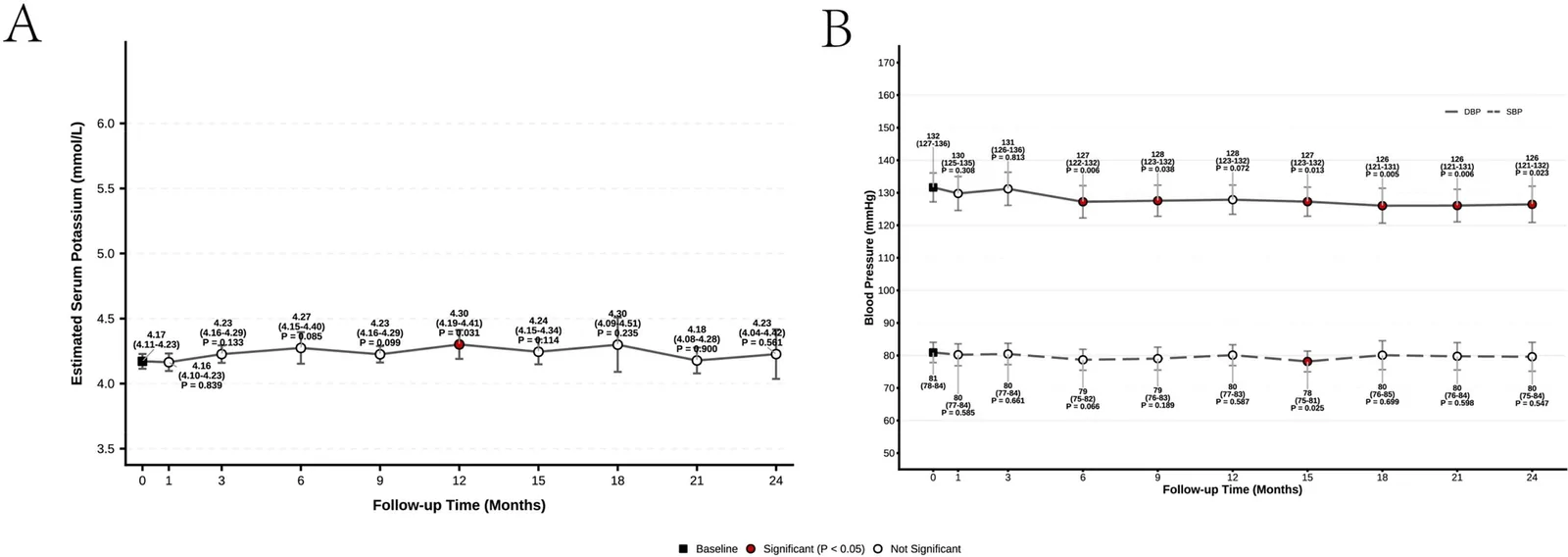
Temporal changes in serum potassium and blood pressure following finerenone initiation in the overall cohort. **(A)** Estimated marginal means of serum potassium over 24 months. **(B)** Estimated marginal means of systolic blood pressure (SBP, solid line) and diastolic blood pressure (DBP, dashed line). Data were derived from Gaussian-identity GEE models with time as a categorical variable and baseline as the reference. Red filled circles indicate statistically significant changes (*P* < 0.05) compared with baseline (black square), while open circles indicate nonsignificant changes. Error bars represent 95% confidence intervals.

## Discussion

In this 24-month real-world cohort of patients with non-diabetic CKD, finerenone initiation was associated with improvement across three complementary kidney efficacy outcomes. UACR declined rapidly and remained significantly below baseline through Month 21, although the Month 24 estimate was less precise and no longer statistically significant. Total urinary protein excretion, measured by 24 hUP, was significantly reduced from Month 3 through Month 24. In parallel, the annualized eGFR slope attenuated from −6.51 mL/min/1.73 m^2^/year before finerenone initiation to −1.44 mL/min/1.73 m^2^/year afterward, corresponding to a significant slope improvement of 5.07 mL/min/1.73 m^2^/year. Collectively, these findings indicate a consistent antiproteinuric association accompanied by a potentially favorable change in kidney-function trajectory.

The concurrent assessment of UACR and 24 hUP provides complementary clinical information. UACR is a standardized measure of albuminuria, and treatment-induced reductions in albuminuria have been associated with a lower subsequent risk of kidney disease progression (6, 24). In contrast, 24 hUP captures total urinary protein excretion and was directly incorporated into the definitions of complete and partial remission used in this cohort. The concordant direction of changes in UACR and 24 hUP supports the internal consistency of the observed antiproteinuric association. The Month-24 UACR estimate was imprecise, with a wide confidence interval in the setting of limited late follow-up; the nonsignificant result should therefore not be interpreted as definitive evidence of loss of response. Because remission definitions are primarily derived from proteinuric glomerular disease studies, their interpretation should be cautious when applied to this heterogeneous non-diabetic CKD population. Therefore, the remission outcomes in this study should be considered indicators of clinically meaningful proteinuria improvement and kidney-function stabilization rather than disease-specific remission states.

The magnitude and direction of albuminuria reduction are broadly consistent with the kidney effects reported in the FIND-CKD trial and with smaller real-world studies of finerenone in non-diabetic CKD (17–19). Recent meta-analytic evidence further supports the potential of combined SGLT-2i and aldosterone/mineralocorticoid receptor-targeted therapy for albuminuria reduction, with no significant increase in serious adverse events (25). The present analysis extends these observations by following a heterogeneous real-world cohort for up to 24 months and by evaluating UACR and 24 hUP concurrently. Nevertheless, direct numerical comparisons should be made cautiously because this study lacked a placebo group, involved different underlying kidney diseases, and used routine-practice follow-up rather than a protocol-driven trial schedule.

The attenuation of the annualized eGFR decline represents an important complementary finding. eGFR slope summarizes kidney-function change over time and is increasingly used as a surrogate endpoint in CKD studies (26–28). In this cohort, the post-treatment slope was significantly less negative than the pre-treatment slope. However, treatment-period vs. pre-treatment-period comparisons in a single-arm retrospective cohort may be influenced by regression to the mean, changes in background therapy, variations in disease activity, informative follow-up, and other time-varying factors. The observed slope improvement should therefore be interpreted as an association with finerenone initiation rather than definitive evidence of a causal treatment effect.

Exploratory subgroup analyses evaluated the consistency of the three efficacy outcomes across clinically relevant patient characteristics. UACR and 24 hUP responses were examined according to baseline eGFR, immunosuppressant use, RASi use, and SGLT-2i use, while the eGFR slope analysis additionally included baseline UACR category. Reductions or slope improvements were observed within several subgroup levels; however, statistical significance within one subgroup and nonsignificance within another do not, by themselves, demonstrate a between-group difference. After Benjamini–Hochberg FDR correction, only the global time-by-RASi interaction for UACR remained significant. This finding suggests that the longitudinal UACR trajectories differed according to baseline RASi use, but the relatively small RASi-negative subgroup and the absence of a consistently larger response in one stratum across all visits limit its clinical interpretation. No interaction for 24 hUP or eGFR slope remained significant after FDR correction, providing no robust evidence that these responses differed according to the evaluated baseline characteristics.

Finerenone was generally well tolerated during the available follow-up. Clinically defined hyperkalemia occurred in 4.96% of participants and was managed with dietary counseling and short-course potassium-lowering treatment, without permanent discontinuation. No serious adverse events were recorded during the available follow-up, and neither hyperkalemia nor hypotension resulted in permanent treatment discontinuation. Longitudinal estimates of serum potassium and blood pressure remained within clinically acceptable ranges. The predominantly low starting dose of finerenone and limited dose escalation may have contributed to the observed tolerability, although conservative dosing may also have reduced the magnitude of the observed efficacy associations. These safety observations are broadly consistent with the manageable hyperkalemia profile reported in previous studies of finerenone (17, 29, 30).

Several limitations should be considered. First, the retrospective, single-center, single-arm design limits causal inference, and residual confounding related to disease etiology and baseline disease severity cannot be completely excluded despite adjustment and sensitivity analyses. The relatively modest sample size (*n* = 121) and predominance of IgA nephropathy may limit generalizability. Second, eligibility incorporated a ≥90-day finerenone requirement. Among patients who did not meet or could not be confirmed to meet this requirement, some had no documented follow-up at our institution within 90 days, and whether treatment was discontinued or continued elsewhere could not be reliably determined. This requirement may introduce selection or survivor bias and limits inference regarding very early treatment discontinuation and safety. Third, because of rolling enrollment, potential follow-up duration varied across patients. At later visits, unavailable data reflected both administrative censoring among patients who had not yet reached the corresponding visit window by database closure and missing outcome data among calendar-eligible patients. Consequently, Month-24 estimates were based on relatively few observations and should be interpreted cautiously. Fourth, subgroup analyses were exploratory and may have been underpowered. Finally, changes in concomitant medications and disease-specific treatments during follow-up may not have been fully captured.

Finerenone initiation was associated with concordant improvements in albuminuria, total proteinuria, and eGFR trajectory, with no major safety signals recorded during the available follow-up in patients with non-diabetic CKD.

## Data Availability

The datasets presented in this article are not readily available because access is restricted by patient privacy protection requirements and hospital data governance rules. Identifiable clinical data are confidential and are not publicly available. The study data are archived internally, and access requires a formal institutional application and approval. Requests to access the datasets should be directed to XuejuanSun@outlook.com.
