# Supplementary material for: Longitudinal kidney outcomes and safety after finerenone initiation in patients with non-diabetic chronic kidney disease: a single-center retrospective cohort study
Source: Front Med (Lausanne). 2026 Oct 19;13:1959259. doi: 10.3389/fmed.2026.1959259 (PMC13724480; doi:10.3389/fmed.2026.1959259)
Supplement: Supplementary file 1 [file Supplementaryfile1.docx]

**SUPPLEMENTARY MATERIAL**

Supplementary Figure S1. Flow diagram of patient selection for the final analytic cohort

Patients remaining (n=141)

Excluded: n=20

• Other prespecified exclusions:

– Substantial missing baseline or follow-up data: n=18

– Baseline hyperkalemia: n=2

– Severe heart failure/uncontrolled hypertension/cirrhosis: n=0

– Other eligibility criteria: n=0

Included in the final analytic cohort (n=121)

Excluded: n=36

• Insufficient 90-day finerenone observation:

– No subsequent documented encounter at our institution within 90 days: n=32

– Treatment initiated <90 days before the database cutoff: n=4

Patients assessed for remaining eligibility (n=181)

Excluded: n=4

• Renal replacement therapy (dialysis or transplantation)

Patients remaining (n=177)

Excluded: n=431

• Type 1 or type 2 diabetes, or baseline HbA1c ≥6.5%

Patients with outpatient finerenone prescriptions screened (n=612)
