# Supplementary material for: Longitudinal kidney outcomes and safety after finerenone initiation in patients with non-diabetic chronic kidney disease: a single-center retrospective cohort study
Source: Front Med (Lausanne). 2026 Oct 19;13:1959259. doi: 10.3389/fmed.2026.1959259 (PMC13724480; doi:10.3389/fmed.2026.1959259)
Supplement: Supplementary file 2 [file Supplementaryfile2.docx]

**SUPPLEMENTARY MATERIAL**

Longitudinal Kidney Outcomes and Safety After Finerenone Initiation in Patients With Non-Diabetic Chronic Kidney Disease: A Single-Center Retrospective Cohort Study

**Supplementary Table S1. Complete subgroup analyses of 24-hour urinary protein after finerenone initiation**

| **Subgroup factor** | **Category** | **Month** | **Adjusted change from baseline, % (95% CI)** | **P value** | **P overall** | **P for interaction** | **FDR-adjusted P** |
| --- | --- | --- | --- | --- | --- | --- | --- |
| Baseline eGFR | <60 | 1 | -14.8 (-26.2 to -1.7) | **0.028** | 0.103 | 0.100 | 0.199 |
| Baseline eGFR | <60 | 3 | -21.9 (-42.0 to 5.2) | 0.104 | 0.103 | 0.100 | 0.199 |
| Baseline eGFR | <60 | 6 | -30.8 (-48.5 to -6.9) | **0.015** | 0.103 | 0.100 | 0.199 |
| Baseline eGFR | <60 | 9 | -25.7 (-50.3 to 11.1) | 0.148 | 0.103 | 0.100 | 0.199 |
| Baseline eGFR | <60 | 12 | -23.9 (-43.0 to 1.5) | 0.063 | 0.103 | 0.100 | 0.199 |
| Baseline eGFR | <60 | 15 | -24.2 (-47.5 to 9.6) | 0.141 | 0.103 | 0.100 | 0.199 |
| Baseline eGFR | <60 | 18 | -26.4 (-50.0 to 8.3) | 0.120 | 0.103 | 0.100 | 0.199 |
| Baseline eGFR | <60 | 21 | -11.5 (-41.0 to 32.8) | 0.555 | 0.103 | 0.100 | 0.199 |
| Baseline eGFR | <60 | 24 | -23.4 (-51.8 to 21.7) | 0.258 | 0.103 | 0.100 | 0.199 |
| Baseline eGFR | ≥60 | 1 | -4.5 (-17.4 to 10.4) | 0.535 | **0.008** | 0.100 | 0.199 |
| Baseline eGFR | ≥60 | 3 | -21.7 (-34.8 to -5.9) | **0.009** | **0.008** | 0.100 | 0.199 |
| Baseline eGFR | ≥60 | 6 | -24.8 (-37.6 to -9.4) | **0.003** | **0.008** | 0.100 | 0.199 |
| Baseline eGFR | ≥60 | 9 | -35.2 (-48.6 to -18.4) | **<0.001** | **0.008** | 0.100 | 0.199 |
| Baseline eGFR | ≥60 | 12 | -40.1 (-53.4 to -23.1) | **<0.001** | **0.008** | 0.100 | 0.199 |
| Baseline eGFR | ≥60 | 15 | -46.9 (-61.3 to -27.2) | **<0.001** | **0.008** | 0.100 | 0.199 |
| Baseline eGFR | ≥60 | 18 | -41.3 (-64.7 to -2.4) | **0.040** | **0.008** | 0.100 | 0.199 |
| Baseline eGFR | ≥60 | 21 | -52.0 (-67.3 to -29.6) | **<0.001** | **0.008** | 0.100 | 0.199 |
| Baseline eGFR | ≥60 | 24 | -49.2 (-72.7 to -5.5) | **0.032** | **0.008** | 0.100 | 0.199 |
| Immunosuppressant | No | 1 | -2.6 (-15.4 to 12.1) | 0.712 | 0.273 | 0.436 | 0.436 |
| Immunosuppressant | No | 3 | -16.6 (-29.6 to -1.3) | **0.035** | 0.273 | 0.436 | 0.436 |
| Immunosuppressant | No | 6 | -21.0 (-35.1 to -3.9) | **0.018** | 0.273 | 0.436 | 0.436 |
| Immunosuppressant | No | 9 | -28.1 (-43.2 to -8.9) | **0.006** | 0.273 | 0.436 | 0.436 |
| Immunosuppressant | No | 12 | -31.9 (-46.5 to -13.2) | **0.002** | 0.273 | 0.436 | 0.436 |
| Immunosuppressant | No | 15 | -33.5 (-53.7 to -4.6) | **0.027** | 0.273 | 0.436 | 0.436 |
| Immunosuppressant | No | 18 | -31.3 (-55.0 to 5.0) | 0.083 | 0.273 | 0.436 | 0.436 |
| Immunosuppressant | No | 21 | -33.4 (-53.1 to -5.4) | **0.023** | 0.273 | 0.436 | 0.436 |
| Immunosuppressant | No | 24 | -34.2 (-57.9 to 2.8) | 0.066 | 0.273 | 0.436 | 0.436 |
| Immunosuppressant | Yes | 1 | -16.9 (-30.6 to -0.4) | **0.045** | **<0.001** | 0.436 | 0.436 |
| Immunosuppressant | Yes | 3 | -25.6 (-47.2 to 4.7) | 0.090 | **<0.001** | 0.436 | 0.436 |
| Immunosuppressant | Yes | 6 | -36.0 (-52.5 to -13.7) | **0.003** | **<0.001** | 0.436 | 0.436 |
| Immunosuppressant | Yes | 9 | -38.4 (-58.4 to -8.8) | **0.015** | **<0.001** | 0.436 | 0.436 |
| Immunosuppressant | Yes | 12 | -39.4 (-60.3 to -7.6) | **0.020** | **<0.001** | 0.436 | 0.436 |
| Immunosuppressant | Yes | 15 | -40.5 (-61.6 to -8.1) | **0.019** | **<0.001** | 0.436 | 0.436 |
| Immunosuppressant | Yes | 18 | -20.2 (-53.8 to 37.7) | 0.417 | **<0.001** | 0.436 | 0.436 |
| Immunosuppressant | Yes | 21 | -36.2 (-64.6 to 14.9) | 0.134 | **<0.001** | 0.436 | 0.436 |
| Immunosuppressant | Yes | 24 | 35.3 (-73.9 to 602.0) | 0.719 | **<0.001** | 0.436 | 0.436 |
| RASi | No | 1 | -16.2 (-30.5 to 1.1) | 0.065 | 0.144 | 0.065 | 0.199 |
| RASi | No | 3 | -18.6 (-36.0 to 3.6) | 0.095 | 0.144 | 0.065 | 0.199 |
| RASi | No | 6 | -29.7 (-50.5 to -0.3) | **0.048** | 0.144 | 0.065 | 0.199 |
| RASi | No | 9 | -34.5 (-58.6 to 3.7) | 0.071 | 0.144 | 0.065 | 0.199 |
| RASi | No | 12 | -5.6 (-40.0 to 48.7) | 0.804 | 0.144 | 0.065 | 0.199 |
| RASi | No | 15 | 31.5 (-36.7 to 173.2) | 0.462 | 0.144 | 0.065 | 0.199 |
| RASi | No | 18 | -45.3 (-92.9 to 323.0) | 0.563 | 0.144 | 0.065 | 0.199 |
| RASi | No | 21 | 10.4 (-95.4 to 2555.2) | 0.951 | 0.144 | 0.065 | 0.199 |
| RASi | No | 24 | -46.6 (-88.8 to 154.3) | 0.431 | 0.144 | 0.065 | 0.199 |
| RASi | Yes | 1 | -5.8 (-17.3 to 7.2) | 0.366 | **0.017** | 0.065 | 0.199 |
| RASi | Yes | 3 | -21.1 (-33.9 to -5.8) | **0.009** | **0.017** | 0.065 | 0.199 |
| RASi | Yes | 6 | -25.7 (-38.0 to -11.0) | **0.001** | **0.017** | 0.065 | 0.199 |
| RASi | Yes | 9 | -32.3 (-45.9 to -15.3) | **<0.001** | **0.017** | 0.065 | 0.199 |
| RASi | Yes | 12 | -38.1 (-50.5 to -22.6) | **<0.001** | **0.017** | 0.065 | 0.199 |
| RASi | Yes | 15 | -40.2 (-55.2 to -20.2) | **<0.001** | **0.017** | 0.065 | 0.199 |
| RASi | Yes | 18 | -36.5 (-56.8 to -6.6) | **0.021** | **0.017** | 0.065 | 0.199 |
| RASi | Yes | 21 | -37.7 (-55.3 to -13.2) | **0.005** | **0.017** | 0.065 | 0.199 |
| RASi | Yes | 24 | -37.1 (-59.3 to -2.8) | **0.037** | **0.017** | 0.065 | 0.199 |
| SGLT-2i | No | 1 | -2.5 (-19.4 to 18.1) | 0.798 | **0.043** | 0.205 | 0.273 |
| SGLT-2i | No | 3 | -14.4 (-32.7 to 8.8) | 0.204 | **0.043** | 0.205 | 0.273 |
| SGLT-2i | No | 6 | -22.2 (-37.5 to -3.1) | **0.025** | **0.043** | 0.205 | 0.273 |
| SGLT-2i | No | 9 | -32.9 (-49.6 to -10.7) | **0.006** | **0.043** | 0.205 | 0.273 |
| SGLT-2i | No | 12 | -34.9 (-50.1 to -15.2) | **0.002** | **0.043** | 0.205 | 0.273 |
| SGLT-2i | No | 15 | -28.1 (-51.2 to 6.0) | 0.096 | **0.043** | 0.205 | 0.273 |
| SGLT-2i | No | 18 | -16.8 (-48.1 to 33.6) | 0.447 | **0.043** | 0.205 | 0.273 |
| SGLT-2i | No | 21 | -31.3 (-53.4 to 1.3) | 0.058 | **0.043** | 0.205 | 0.273 |
| SGLT-2i | No | 24 | -29.2 (-59.8 to 24.7) | 0.232 | **0.043** | 0.205 | 0.273 |
| SGLT-2i | Yes | 1 | -10.2 (-22.0 to 3.4) | 0.135 | **0.008** | 0.205 | 0.273 |
| SGLT-2i | Yes | 3 | -23.4 (-34.7 to -10.2) | **0.001** | **0.008** | 0.205 | 0.273 |
| SGLT-2i | Yes | 6 | -29.0 (-45.0 to -8.3) | **0.009** | **0.008** | 0.205 | 0.273 |
| SGLT-2i | Yes | 9 | -28.1 (-45.6 to -4.9) | **0.021** | **0.008** | 0.205 | 0.273 |
| SGLT-2i | Yes | 12 | -30.6 (-49.4 to -5.0) | **0.023** | **0.008** | 0.205 | 0.273 |
| SGLT-2i | Yes | 15 | -43.5 (-62.5 to -14.7) | **0.007** | **0.008** | 0.205 | 0.273 |
| SGLT-2i | Yes | 18 | -48.1 (-66.0 to -20.7) | **0.002** | **0.008** | 0.205 | 0.273 |
| SGLT-2i | Yes | 21 | -42.9 (-62.0 to -14.3) | **0.007** | **0.008** | 0.205 | 0.273 |
| SGLT-2i | Yes | 24 | -41.2 (-65.1 to -1.0) | **0.046** | **0.008** | 0.205 | 0.273 |

Values are adjusted percentage changes from baseline with 95% CIs from gamma-log GEE models with an AR(1) working correlation structure and robust sandwich standard errors. Models were adjusted for age, sex, RASi use, SGLT-2i use, and immunosuppressant use; the stratifying variable was omitted from the corresponding subgroup model. P overall is the global test for change across follow-up within each subgroup level. P for interaction is the global time-by-subgroup interaction test across all nine post-baseline visits. FDR adjustment used the Benjamini-Hochberg method across the four 24hUP interaction tests.

**Supplementary Table S2. Complete subgroup analyses of urinary albumin-to-creatinine ratio after finerenone initiation**

| **Subgroup factor** | **Category** | **Month** | **Adjusted change from baseline, % (95% CI)** | **P value** | **P overall** | **P for interaction** | **FDR-adjusted P** |
| --- | --- | --- | --- | --- | --- | --- | --- |
| Baseline eGFR | <60 | 1 | -26.8 (-45.0 to -2.5) | **0.033** | **0.004** | 0.092 | 0.184 |
| Baseline eGFR | <60 | 3 | -36.9 (-53.7 to -14.1) | **0.003** | **0.004** | 0.092 | 0.184 |
| Baseline eGFR | <60 | 6 | -50.3 (-65.4 to -28.7) | **<0.001** | **0.004** | 0.092 | 0.184 |
| Baseline eGFR | <60 | 9 | -42.8 (-60.3 to -17.7) | **0.003** | **0.004** | 0.092 | 0.184 |
| Baseline eGFR | <60 | 12 | -42.9 (-58.8 to -20.9) | **<0.001** | **0.004** | 0.092 | 0.184 |
| Baseline eGFR | <60 | 15 | -36.3 (-55.9 to -8.0) | **0.016** | **0.004** | 0.092 | 0.184 |
| Baseline eGFR | <60 | 18 | -37.0 (-57.0 to -7.9) | **0.017** | **0.004** | 0.092 | 0.184 |
| Baseline eGFR | <60 | 21 | -27.8 (-56.2 to 18.9) | 0.201 | **0.004** | 0.092 | 0.184 |
| Baseline eGFR | <60 | 24 | -15.6 (-45.9 to 31.8) | 0.456 | **0.004** | 0.092 | 0.184 |
| Baseline eGFR | ≥60 | 1 | -23.0 (-33.6 to -10.7) | **<0.001** | **<0.001** | 0.092 | 0.184 |
| Baseline eGFR | ≥60 | 3 | -44.7 (-55.2 to -31.8) | **<0.001** | **<0.001** | 0.092 | 0.184 |
| Baseline eGFR | ≥60 | 6 | -40.3 (-53.8 to -22.9) | **<0.001** | **<0.001** | 0.092 | 0.184 |
| Baseline eGFR | ≥60 | 9 | -47.2 (-61.4 to -27.7) | **<0.001** | **<0.001** | 0.092 | 0.184 |
| Baseline eGFR | ≥60 | 12 | -51.4 (-64.7 to -33.1) | **<0.001** | **<0.001** | 0.092 | 0.184 |
| Baseline eGFR | ≥60 | 15 | -57.0 (-70.0 to -38.4) | **<0.001** | **<0.001** | 0.092 | 0.184 |
| Baseline eGFR | ≥60 | 18 | -39.9 (-67.5 to 11.2) | 0.105 | **<0.001** | 0.092 | 0.184 |
| Baseline eGFR | ≥60 | 21 | -54.1 (-70.8 to -27.8) | **<0.001** | **<0.001** | 0.092 | 0.184 |
| Baseline eGFR | ≥60 | 24 | -47.6 (-80.6 to 41.5) | 0.202 | **<0.001** | 0.092 | 0.184 |
| Immunosuppressant | No | 1 | -24.2 (-34.8 to -11.8) | **<0.001** | **<0.001** | 0.963 | 0.963 |
| Immunosuppressant | No | 3 | -40.8 (-51.7 to -27.3) | **<0.001** | **<0.001** | 0.963 | 0.963 |
| Immunosuppressant | No | 6 | -40.0 (-53.2 to -23.1) | **<0.001** | **<0.001** | 0.963 | 0.963 |
| Immunosuppressant | No | 9 | -43.9 (-58.6 to -24.0) | **<0.001** | **<0.001** | 0.963 | 0.963 |
| Immunosuppressant | No | 12 | -47.4 (-60.8 to -29.3) | **<0.001** | **<0.001** | 0.963 | 0.963 |
| Immunosuppressant | No | 15 | -48.3 (-64.8 to -24.0) | **<0.001** | **<0.001** | 0.963 | 0.963 |
| Immunosuppressant | No | 18 | -38.8 (-62.5 to -0.2) | **0.049** | **<0.001** | 0.963 | 0.963 |
| Immunosuppressant | No | 21 | -40.3 (-61.8 to -6.7) | **0.023** | **<0.001** | 0.963 | 0.963 |
| Immunosuppressant | No | 24 | -34.0 (-64.9 to 24.1) | 0.197 | **<0.001** | 0.963 | 0.963 |
| Immunosuppressant | Yes | 1 | -12.3 (-25.7 to 3.7) | 0.125 | **0.003** | 0.963 | 0.963 |
| Immunosuppressant | Yes | 3 | -32.1 (-50.0 to -7.7) | **0.013** | **0.003** | 0.963 | 0.963 |
| Immunosuppressant | Yes | 6 | -41.7 (-59.3 to -16.5) | **0.003** | **0.003** | 0.963 | 0.963 |
| Immunosuppressant | Yes | 9 | -44.7 (-61.8 to -20.0) | **0.002** | **0.003** | 0.963 | 0.963 |
| Immunosuppressant | Yes | 12 | -47.2 (-62.7 to -25.3) | **<0.001** | **0.003** | 0.963 | 0.963 |
| Immunosuppressant | Yes | 15 | -42.8 (-64.6 to -7.8) | **0.022** | **0.003** | 0.963 | 0.963 |
| Immunosuppressant | Yes | 18 | -26.4 (-58.6 to 31.0) | 0.298 | **0.003** | 0.963 | 0.963 |
| Immunosuppressant | Yes | 21 | -33.9 (-65.0 to 24.6) | 0.200 | **0.003** | 0.963 | 0.963 |
| Immunosuppressant | Yes | 24 | -20.9 (-54.3 to 37.0) | 0.403 | **0.003** | 0.963 | 0.963 |
| RASi | No | 1 | -36.0 (-53.9 to -11.2) | **0.008** | **<0.001** | **<0.001** | **<0.001** |
| RASi | No | 3 | -30.4 (-56.6 to 11.6) | 0.133 | **<0.001** | **<0.001** | **<0.001** |
| RASi | No | 6 | -50.4 (-69.9 to -18.5) | **0.006** | **<0.001** | **<0.001** | **<0.001** |
| RASi | No | 9 | -57.2 (-78.4 to -15.5) | **0.015** | **<0.001** | **<0.001** | **<0.001** |
| RASi | No | 12 | -51.5 (-79.2 to 13.1) | 0.094 | **<0.001** | **<0.001** | **<0.001** |
| RASi | No | 15 | -50.7 (-82.6 to 40.1) | 0.185 | **<0.001** | **<0.001** | **<0.001** |
| RASi | No | 18 | -42.9 (-74.7 to 29.0) | 0.178 | **<0.001** | **<0.001** | **<0.001** |
| RASi | No | 21 | -66.2 (-84.7 to -25.3) | **0.007** | **<0.001** | **<0.001** | **<0.001** |
| RASi | No | 24 | -33.8 (-62.1 to 15.4) | 0.146 | **<0.001** | **<0.001** | **<0.001** |
| RASi | Yes | 1 | -19.4 (-29.0 to -8.4) | **<0.001** | **<0.001** | **<0.001** | **<0.001** |
| RASi | Yes | 3 | -41.8 (-50.6 to -31.4) | **<0.001** | **<0.001** | **<0.001** | **<0.001** |
| RASi | Yes | 6 | -38.5 (-50.4 to -23.7) | **<0.001** | **<0.001** | **<0.001** | **<0.001** |
| RASi | Yes | 9 | -42.3 (-54.8 to -26.3) | **<0.001** | **<0.001** | **<0.001** | **<0.001** |
| RASi | Yes | 12 | -47.0 (-57.8 to -33.4) | **<0.001** | **<0.001** | **<0.001** | **<0.001** |
| RASi | Yes | 15 | -48.4 (-60.7 to -32.2) | **<0.001** | **<0.001** | **<0.001** | **<0.001** |
| RASi | Yes | 18 | -37.0 (-59.3 to -2.5) | **0.038** | **<0.001** | **<0.001** | **<0.001** |
| RASi | Yes | 21 | -37.2 (-56.7 to -8.8) | **0.015** | **<0.001** | **<0.001** | **<0.001** |
| RASi | Yes | 24 | -33.4 (-63.4 to 21.0) | 0.182 | **<0.001** | **<0.001** | **<0.001** |
| SGLT-2i | No | 1 | -26.6 (-38.8 to -12.0) | **<0.001** | **<0.001** | 0.208 | 0.278 |
| SGLT-2i | No | 3 | -37.4 (-50.0 to -21.6) | **<0.001** | **<0.001** | 0.208 | 0.278 |
| SGLT-2i | No | 6 | -39.7 (-52.9 to -22.8) | **<0.001** | **<0.001** | 0.208 | 0.278 |
| SGLT-2i | No | 9 | -41.7 (-59.3 to -16.4) | **0.003** | **<0.001** | 0.208 | 0.278 |
| SGLT-2i | No | 12 | -46.5 (-62.1 to -24.5) | **<0.001** | **<0.001** | 0.208 | 0.278 |
| SGLT-2i | No | 15 | -41.2 (-62.4 to -8.1) | **0.020** | **<0.001** | 0.208 | 0.278 |
| SGLT-2i | No | 18 | -22.5 (-58.0 to 43.1) | 0.416 | **<0.001** | 0.208 | 0.278 |
| SGLT-2i | No | 21 | -42.1 (-70.0 to 11.7) | 0.103 | **<0.001** | 0.208 | 0.278 |
| SGLT-2i | No | 24 | -21.2 (-63.4 to 69.5) | 0.541 | **<0.001** | 0.208 | 0.278 |
| SGLT-2i | Yes | 1 | -19.8 (-32.4 to -4.7) | **0.012** | **0.002** | 0.208 | 0.278 |
| SGLT-2i | Yes | 3 | -42.9 (-55.9 to -26.2) | **<0.001** | **0.002** | 0.208 | 0.278 |
| SGLT-2i | Yes | 6 | -43.9 (-60.9 to -19.6) | **0.002** | **0.002** | 0.208 | 0.278 |
| SGLT-2i | Yes | 9 | -49.7 (-64.8 to -28.0) | **<0.001** | **0.002** | 0.208 | 0.278 |
| SGLT-2i | Yes | 12 | -50.6 (-65.6 to -29.0) | **<0.001** | **0.002** | 0.208 | 0.278 |
| SGLT-2i | Yes | 15 | -57.1 (-72.0 to -34.3) | **<0.001** | **0.002** | 0.208 | 0.278 |
| SGLT-2i | Yes | 18 | -57.0 (-71.3 to -35.7) | **<0.001** | **0.002** | 0.208 | 0.278 |
| SGLT-2i | Yes | 21 | -44.1 (-63.8 to -13.7) | **0.009** | **0.002** | 0.208 | 0.278 |
| SGLT-2i | Yes | 24 | -63.8 (-78.1 to -40.1) | **<0.001** | **0.002** | 0.208 | 0.278 |

Values are adjusted percentage changes from baseline with 95% CIs from gamma-log GEE models with an AR(1) working correlation structure and robust sandwich standard errors. Models were adjusted for age, sex, RASi use, SGLT-2i use, and immunosuppressant use; the stratifying variable was omitted from the corresponding subgroup model. P overall is the global test for change across follow-up within each subgroup level. P for interaction is the global time-by-subgroup interaction test across all nine post-baseline visits. FDR adjustment used the Benjamini-Hochberg method across the four UACR interaction tests.

**Supplementary Table S3. Extended-adjustment sensitivity analysis of UACR and 24-hour urinary protein**

| Outcome | Month | Adjusted change from baseline, % (95% CI) | P value |
| --- | --- | --- | --- |
| UACR | 1 | -22.2 (-31.0 to -12.2) | <0.001 |
| UACR | 3 | -40.0 (-49.0 to -29.3) | <0.001 |
| UACR | 6 | -40.7 (-51.3 to -27.8) | <0.001 |
| UACR | 9 | -42.6 (-54.8 to -27.1) | <0.001 |
| UACR | 12 | -47.2 (-58.2 to -33.3) | <0.001 |
| UACR | 15 | -48.8 (-61.0 to -32.9) | <0.001 |
| UACR | 18 | -36.7 (-59.5 to -1.0) | 0.045 |
| UACR | 21 | -42.2 (-59.0 to -18.5) | 0.002 |
| UACR | 24 | -36.8 (-63.9 to 10.8) | 0.109 |
| 24hUP | 1 | -6.3 (-16.6 to 5.2) | 0.269 |
| 24hUP | 3 | -19.8 (-31.1 to -6.6) | 0.005 |
| 24hUP | 6 | -24.6 (-35.3 to -12.3) | <0.001 |
| 24hUP | 9 | -30.0 (-42.7 to -14.6) | <0.001 |
| 24hUP | 12 | -35.1 (-46.6 to -21.1) | <0.001 |
| 24hUP | 15 | -36.9 (-51.1 to -18.5) | <0.001 |
| 24hUP | 18 | -31.5 (-51.7 to -2.9) | 0.033 |
| 24hUP | 21 | -36.5 (-51.7 to -16.4) | 0.001 |
| 24hUP | 24 | -35.6 (-56.7 to -4.1) | 0.030 |

Values are adjusted percentage changes from baseline with 95% CIs from gamma-log GEE models using an AR(1) working correlation structure and robust sandwich standard errors. Models additionally adjusted for baseline eGFR (continuous) and kidney disease etiology (IgA nephropathy, membranous nephropathy, or other), in addition to age, sex, RASi use, SGLT-2i use, and immunosuppressant use. Analysis samples: UACR, 112 patients/742 observations; 24hUP, 112 patients/737 observations. Global time effect: UACR, P<0.001; 24hUP, P=0.005. Abbreviations: 24hUP, 24-hour urinary protein; GEE, generalized estimating equation; UACR, urinary albumin-to-creatinine ratio.

**Supplementary Table S4. Sensitivity analyses of annualized eGFR slopes**

| Model | Patients / observations | Pre-treatment slope, estimate (95% CI); P | Post-treatment slope, estimate (95% CI); P | Slope improvement, estimate (95% CI); P |
| --- | --- | --- | --- | --- |
| Reference primary eGFR LMM | 115 / 880 | -6.51 (-9.70 to -3.31); P<0.001 | -1.44 (-3.52 to 0.64); P=0.174 | 5.07 (1.86 to 8.27); P=0.002 |
| Sensitivity 1A: + kidney disease etiology | 115 / 880 | -6.49 (-9.69 to -3.30); P<0.001 | -1.45 (-3.53 to 0.63); P=0.170 | 5.04 (1.84 to 8.25); P=0.002 |
| Sensitivity 1B: etiology + continuous log baseline UACR | 112 / 859 | -6.56 (-9.79 to -3.33); P<0.001 | -1.26 (-3.39 to 0.88); P=0.246 | 5.30 (2.09 to 8.52); P=0.001 |

Slopes are expressed as mL/min/1.73 m²/year. Slope improvement is the post-treatment slope minus the pre-treatment slope. The reference model adjusted for age, sex, baseline UACR category, RASi use, SGLT-2i use, and immunosuppressant use. Sensitivity 1A additionally adjusted for kidney disease etiology. Sensitivity 1B adjusted for kidney disease etiology and replaced categorical baseline UACR with continuous log-transformed baseline UACR; three patients without continuous baseline UACR were therefore excluded from this complete-case model. All models were segmented linear mixed-effects models with patient-specific random intercepts and random slopes, fitted by restricted maximum likelihood with Satterthwaite-based inference. None of the models was singular. Abbreviations: LMM, linear mixed-effects model; UACR, urinary albumin-to-creatinine ratio.

**Supplementary Table S5. Post-baseline proteinuria sensitivity analysis explicitly adjusting for baseline proteinuria**

| **Outcome** | **Month** | **Standardized change from common baseline reference, % (95% CI)** | **Standardized baseline geometric mean** |
| --- | --- | --- | --- |
| UACR | 1 | -0.1 (-10.6 to 11.6) | 527.2 |
| UACR | 3 | -17.0 (-27.0 to -5.5) | 527.2 |
| UACR | 6 | -19.3 (-30.8 to -6.0) | 527.2 |
| UACR | 9 | -9.0 (-28.3 to 15.5) | 527.2 |
| UACR | 12 | -23.6 (-35.5 to -9.6) | 527.2 |
| UACR | 15 | -17.5 (-35.5 to 5.6) | 527.2 |
| UACR | 18 | -0.6 (-34.3 to 50.4) | 527.2 |
| UACR | 21 | -22.1 (-40.7 to 2.3) | 527.2 |
| UACR | 24 | -18.9 (-44.9 to 19.4) | 527.2 |
| 24hUP | 1 | 13.7 (1.2 to 27.8) | 885.7 |
| 24hUP | 3 | 1.5 (-11.5 to 16.5) | 885.7 |
| 24hUP | 6 | -0.9 (-15.6 to 16.4) | 885.7 |
| 24hUP | 9 | -5.4 (-19.1 to 10.6) | 885.7 |
| 24hUP | 12 | -11.9 (-24.5 to 2.9) | 885.7 |
| 24hUP | 15 | -11.5 (-28.1 to 8.9) | 885.7 |
| 24hUP | 18 | -4.2 (-30.2 to 31.5) | 885.7 |
| 24hUP | 21 | -7.9 (-32.0 to 24.7) | 885.7 |
| 24hUP | 24 | -15.2 (-38.1 to 16.2) | 885.7 |

Values are model-based standardized estimates from post-baseline gamma-log GEE models (Months 1-24) adjusted for the corresponding log-transformed baseline proteinuria value, baseline eGFR, kidney disease etiology, age, sex, RASi use, SGLT-2i use, and immunosuppressant use. Standardized estimates were evaluated at the patient-level mean covariate profile and expressed relative to the geometric-mean baseline value (UACR, 527.2 mg/g; 24hUP, 885.7 mg/24 h). These estimates are not directly equivalent to the primary baseline-included GEE estimand. Analysis samples: UACR, 112 patients/630 post-baseline observations; 24hUP, 112 patients/625 post-baseline observations. Abbreviations: 24hUP, 24-hour urinary protein; GEE, generalized estimating equation; UACR, urinary albumin-to-creatinine ratio.

**Supplementary Table S6. Formal post-baseline time-by-immunosuppression interaction after adjustment for baseline proteinuria**

| **Outcome** | **Patients** | **Post-baseline observations** | **Wald χ²** | **df** | **P for time × immunosuppression interaction** |
| --- | --- | --- | --- | --- | --- |
| UACR | 112 | 630 | 8.86 | 8 | 0.354 |
| 24hUP | 112 | 625 | 14.80 | 8 | 0.063 |

The formal interaction test was an 8-df joint Wald test of the Month 3 through Month 24 time-by-immunosuppression interaction coefficients, with Month 1 as the post-baseline reference visit. Models additionally adjusted for the corresponding log-transformed baseline proteinuria value, baseline eGFR, kidney disease etiology, age, sex, RASi use, and SGLT-2i use. The interaction was not statistically significant for UACR (P=0.354) or 24hUP (P=0.063). Abbreviations: 24hUP, 24-hour urinary protein; UACR, urinary albumin-to-creatinine ratio.

**Supplementary Table S7. Follow-up data availability according to calendar eligibility at scheduled visits**

| **Visit** | **Calendar-eligible** | **Key outcome available** | **Missing despite eligibility** | **Not yet due by cutoff** | **Availability among eligible** |
| --- | --- | --- | --- | --- | --- |
| Month1 | 121 | 110 | 11 | 0 | 90.90% |
| Month3 | 121 | 118 | 3 | 0 | 97.50% |
| Month6 | 121 | 118 | 3 | 0 | 97.50% |
| Month9 | 116 | 103 | 13 | 5 | 88.80% |
| Month12 | 103 | 82 | 21 | 18 | 79.60% |
| Month15 | 97 | 60 | 37 | 24 | 61.90% |
| Month18 | 88 | 46 | 42 | 33 | 52.30% |
| Month21 | 73 | 27 | 46 | 48 | 37.00% |
| Month24 | 46 | 20 | 26 | 75 | 43.50% |

The median observed follow-up duration, defined as the interval from finerenone initiation to the last documented encounter, was 434 days (IQR, 278–604 days). Calendar eligibility was determined according to the prespecified visit windows (Month 1, ±15 days; Months 3–24, ±30 days) and a database cutoff of December 31, 2025. Patients classified as “not yet due by cutoff” had insufficient calendar time to enter the corresponding visit window before database closure. “Missing despite eligibility” indicates that the patient had sufficient calendar time to reach the visit window but no key outcome was available within that window. Key outcome availability was defined as availability of at least one of UACR, 24hUP, or scheduled eGFR within the corresponding visit window.

**Supplementary Table S8. Baseline characteristics according to Month-24 key outcome availability among calendar-eligible patients**

| Variable | Month-24 key outcome available (n=20) | Missing despite Month-24 eligibility (n=26) | P value |
| --- | --- | --- | --- |
| Age, years | 46.5 (40.8–55.8) | 48.5 (36.2–57.8) | 0.920 |
| Male sex, n (%) | 7 (35.0%) | 14 (53.8%) | 0.244 |
| Primary renal disease, n (%) |  |  | 0.096 |
| IgA nephropathy | 13 (65.0%) | 9 (34.6%) |  |
| Membranous nephropathy | 4 (20.0%) | 12 (46.2%) |  |
| Other | 3 (15.0%) | 5 (19.2%) |  |
| eGFR, mL/min/1.73 m² | 69.23 (57.87–90.25) | 75.93 (48.52–94.50) | 0.833 |
| eGFR category, n (%) |  |  | 0.514 |
| <30 mL/min/1.73 m² | 1 (5.0%) | 0 (0.0%) |  |
| 30–<60 mL/min/1.73 m² | 5 (25.0%) | 9 (34.6%) |  |
| ≥60 mL/min/1.73 m² | 14 (70.0%) | 17 (65.4%) |  |
| 24-h urinary protein, mg/24 h | 863.0 (416.6–1464.5) | 1398.5 (815.7–2580.9) | 0.032 |
| UACR, mg/g | 544.2 (269.5–1034.6) | 764.2 (494.9–1330.4) | 0.129 |
| UACR ≥300 mg/g, n (%) | 13 (65.0%) | 24 (92.3%) | 0.029 |
| Serum potassium, mmol/L | 4.12 (3.87–4.31) | 4.16 (3.98–4.34) | 0.782 |
| Serum albumin, g/L | 40.30 (37.95–43.20), n=19 | 40.70 (37.50–42.90) | 0.713 |
| ALT, U/L | 17.45 (13.05–24.47), n=14 | 20.30 (15.20–32.80), n=21 | 0.346 |
| AST, U/L | 22.20 (19.12–24.55), n=14 | 24.10 (17.60–26.00), n=21 | 0.736 |
| CK, U/L | 74.5 (58.2–85.5), n=14 | 82.0 (50.0–100.0), n=21 | 0.674 |
| Systolic blood pressure, mmHg | 128.0 (125.0–146.0), n=9 | 137.5 (125.2–144.5), n=12 | 0.722 |
| Diastolic blood pressure, mmHg | 78.0 (72.0–91.0), n=9 | 81.5 (75.0–85.2), n=12 | 0.915 |
| Steroid/immunosuppressive therapy, n (%) | 1 (5.0%) | 9 (34.6%) | 0.028 |
| RAS inhibitor use, n (%) | 17 (85.0%) | 22 (84.6%) | 1.000 |
| SGLT2 inhibitor use, n (%) | 8 (40.0%) | 10 (38.5%) | 1.000 |
| Finerenone starting dose 20 mg, n (%) | 0 (0.0%) | 0 (0.0%) | 1.000 |
